# High‐Performance Industrial‐Grade CsPbBr_3_ Single Crystal by Solid–Liquid Interface Engineering

**DOI:** 10.1002/advs.202302236

**Published:** 2023-06-06

**Authors:** Qihao Sun, Bangzhi Ge, Bao Xiao, Fangpei Li, Leilei Ji, Ziang Yin, Jun Guo, Jia Tang, Chongjian Zhou, Wanqi Jie, Menghua Zhu, Yadong Xu

**Affiliations:** ^1^ State Key Laboratory of Solidification Processing & Key Laboratory of Radiation Detection Materials and Devices MIIT School of Materials Science and Engineering Northwestern Polytechnical University Xi'an 710072 China

**Keywords:** CsPbBr_3_, *γ*‐ray detection, single crystal

## Abstract

All‐inorganic metal halide perovskite CsPbBr_3_ crystal is regarded as an attractive alternative to high purity Ge and CdZnTe for room temperature *γ*‐ray detection. However, high *γ*‐ray resolution is only observable in small CsPbBr_3_ crystal; more practical and deployable large crystal exhibits very low, and even no detection efficiency, thereby thwarting prospects for cost‐effective room temperature *γ*‐ray detection. The poor performance of large crystal is attributed to the unexpected secondary phase inclusion during crystal growth, which traps the generated carriers. Here, the solid–liquid interface during crystal growth is engineered by optimizing the temperature gradient and growth velocity. This minimizes the unfavorable formation of the secondary phase, leading to industrial‐grade crystals with a diameter of 30 mm. This excellent‐quality crystal exhibits remarkably high carrier mobility of 35.4 cm^2^ V^−1^ s^−1^ and resolves the peak of ^137^Cs@ 662 keV *γ*‐ray at an energy resolution of 9.91%. These values are the highest among previously reported large crystals.

## Graphical abstract

All‐inorganic metal halide perovskite CsPbBr_3_ crystal is regarded as a candidate for room temperature *γ*‐ray detection. An industrial‐grade CsPbBr_3_ crystal with diameter of 30 mm exhibits remarkably high carrier mobility of 35.4 cm^2^ V^−1^ s^−1^ and resolves the peak of ^137^Cs@662 keV *γ*‐ray at an energy resolution of 9.91%. These values are the highest among previously reported large crystals.

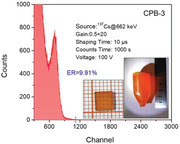

## 1 Introduction

High‐resolution, large‐volume gamma‐ray (*γ*‐ray) detectors play critical roles in homeland security, industrial, and biomedical applications.^[^ ^1^ ^]^ Scintillator and semiconductor‐based detectors dominate the commercial market,^[^ ^2^ ^]^ with thallium‐doped sodium iodide having a 50% share,^[^ ^3^ ^]^ and the remaining half is mostly high‐purity Ge (HPGe) and CdZnTe.^[^ ^4^ ^]^ The former only operates at liquid nitrogen temperature, while the latter can work at room temperature but contains elements of low abundance on earth. Importantly, the process cost of both materials is very high because extremely high purity of 12N and 5N are required for HPGe and CdZnTe,^[^ ^5^ ^]^ respectively. Low‐cost semiconductor materials working at room temperature with high resolution have been a long‐time sought‐goal in this field.

CsPbBr_3_(CPB) is an all‐inorganic metal halide perovskite, which has been predicted to be able to offer transformative advantages in detecting *γ*‐ray because of its large light absorption coefficient, high carrier mobility, and long charge diffusion length.^[^ ^6^ ^]^ The Kanatzidis group reported in 2018 that 3.9% energy resolution for 122 keV ^57^Co *γ*‐ray was obtained at room temperature in CsPbBr_3_ crystal grown by the vertical Bridgman method.^[^ ^7^ ^]^ However, many research groups did not observe any *γ*‐ray signal by CsPbBr_3_.^[^ ^8^ ^]^ This has led to controversy regarding the detection efficiency of *γ*‐ray of CsPbBr_3_ crystal. Consensus has been reached that low detection efficiency arises from unexpected secondary phase, indicating CsPb_2_Br_5_ significantly reduces detection efficiency.^[^ ^9^ ^]^ Despite the composition and morphology of the inclusion of secondary phase in bulk CsPbBr_3_ being thoroughly investigated,^[^ ^9^, ^10^ ^]^ removal of these secondary phases remains elusive. Consequently, it is a huge challenge to synthesis large‐size high‐quality CsPbBr_3_ crystal, which has a realistic chance for commercializing and displacing traditional semiconductor *γ*‐ray detection systems.

To initially mitigate this problem; herein, we established the relationship between the crystal growth process and the secondary phase in CsPbBr_3_ crystal grown by the vertical Bridgman method. CsPbBr_3_ crystals with a diameter of 15 mm were employed to engineer the solid–liquid interface during crystal growth by adjusting the temperature gradient (*G*_*L*_) and crystal growth velocity (*V*). The CsPbBr_3_ crystals were quenched by in situ furnace cooling to freeze the solid–liquid growth interface, revealing the optimized condition for the synthesis of high‐quality crystal nearly free of the secondary phase. Accordingly, we synthesized industrial‐grade CsPbBr_3_ crystal with a diameter of 30 mm, which resolves the peak of ^137^Cs@662 keV *γ*‐ray at an energy resolution of 9.91%.

## 2 Results and Discussion

**Figure 1:**
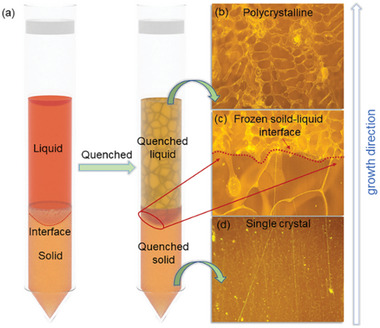
a) Schematic diagram of frozen solid–liquid interface by quenching during the crystal growth using vertical Bridgman furnace. b–d) Represents optical images taken at the bottom, near the solid–liquid interface, and top regions of quenched CsPbBr_3_ sample, respectively.

To pull high‐quality CsPbBr_3_ crystals by vertical Bridgman method, a smooth and stable phase boundary is required. However, the constitutional supercooling can destabilize solid–liquid interface and form a cellular interface, which significantly reduces the crystal quality.^[^ ^5^, ^11^ ^]^ Accordingly, it is important to flatten the phase boundary during crystal growth. To directly visualize the impact of temperature gradient and growth rate on phase boundary, we froze the solid–liquid interface by quenching the sample with a diameter of 15 mm before liquid phase fully solidified into crystal as sketched in **Figure** **1**a. The sample quenched from temperature gradient (*G*_*L*_) of 12.1 K cm^−1^ and growth rate (*V*) of 1.0 mm h^−1^ was set as control sample (denoted as QC‐1). To minimize the interface fluctuation, the *G*_*L*_ and *V* were set to 12.1 K cm^−1^ and 0.5 mm h^−1^ (QC‐2), following with optimized parameters *G*_*L*_ of 25.3 K cm^−1^ and *V* of 0.5 mm h^−1^(QC‐3). The temperature profiles and the *G*_*L*_/*V* ratios for synthesizing CsPbBr_3_ are detailed in the Supporting Information (Figure S1a,b and Table S1, Supporting Information).

Figure 1b displays the cross‐sectional optical images of QC‐1 recorded along its growth direction. The results reveal that the sample forms single crystal in the lower part and abruptly turns into polycrystalline in the upper part, which creates a clear and tightly jointed interface. Given the quench process induces heterogenous nucleation sites, the polycrystalline region in the sample corresponds to the liquid phase. Accordingly, the interface formed between single crystalline and polycrystalline region can be attributed to the solid–liquid interface during crystal growth.

**Figure 2:**
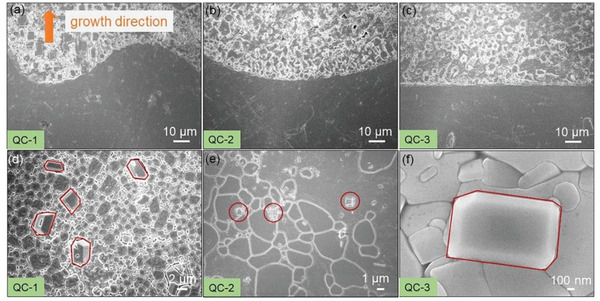
SEM images taken on the solid–liquid interface of a) QC‐1, b) QC‐2, and c) QC‐3, showing the interfaces were gradually flattened. d–f) High‐magnification SEM image taken on the polycrystalline regions of QC‐1, QC‐2, and QC‐3, respectively. The red lines outline the secondary phases.

Subsequently, we further determined the shape of solid–liquid interface by scanning electron microscope (SEM). The sample synthesized with small *G*_L_ and large *V*, namely sample QC‐1, demonstrates a corrugated interface with a periodicity of around half hundred micrometers (**Figure** **2**a; Figure S2a, Supporting Information). Optimizing the synthesis parameter can effectively flatten the distorted phase boundary. For example, QC‐2 sample with a slower growth rate exhibits a periodic fluctuation interface (Figure 2b). It is highly remarkable that simultaneously reducing growth rate and increasing temperature gradient give a nearly flattened interface across the entire sample without any discernible fluctuation (Figure 2c; Figure S2b, Supporting Information).

It is important to note that the secondary phases are observed near the solid–liquid interface regardless of the synthesis recipe. The representative high‐magnification SEM images determine the length of secondary phases in QC‐1, QC‐2, and QC‐3 to be roughly 10, 2, and 1.5 µm as outlined by the red lines, respectively (Figure 2d,e), indicating their sizes are seemingly associated with the shape of interface and that optimizing the synthesis parameters also contributes significantly to cease the unfavorable formation of secondary phase. Importantly, the EDS results in Figure S3, Supporting Information, indicate that the Pb‐rich phases are present at the front of the solid–liquid interface; namely, Pb and Br are enriched at the front of the solid–liquid interface during solidification. The given atomic ratios in Table S1, Supporting Information, confirm that the over‐stoichiometric Pb and Br in the liquid phase further precipitate into the CsPb_2_Br_5_ phases during the quenching process.

These findings can be explained by the constitutional supercooling. In the framework of the liquid phase only involving diffusion, the constitutional supercooling criterion has been theoretically proposed as follows:^[^ ^12^ ^]^

(1) $$\frac{G_{L}}{V}\geq \frac{mC_{0}\left(1-k_{0}\right)}{D_{L}k_{0}}$$

where *G*_*L*_ *and V* denote the temperature gradient near the growth interface on the liquid phase and the growth rate, respectively. *D*_*L*_ represents the diffusion coefficient of the solute, *C*_0_ is the initial solute concentration in the melt, *k*_0_ is the equilibrium partition coefficient of the solute at the growth interface, and *m* is the slope of the liquidus in the phase diagram. Note that secondary phases can be confirmed as Pb‐rich component due to stoichiometry deviation of liquid. We estimated the composition of the liquid phase (polycrystalline) ≈ 17 at% of Cs, and the *m* could be roughly estimated to be ≈1.67 highlighted in phase diagram (Figure S4, Supporting Information).

According to the PbBr_2_−CsBr pseudo binary phase diagram, CsPb_2_Br_5_ is formed under PbBr_2_‐rich condition during the growth. Considering the high melting point of CsBr (909 K), [CsBr]‐clusters tend to be formed in the melt away from the S−L interface due to insufficient superheating. Thus, the accumulation of PbBr_2_‐rich droplets was near the S–L interface. This led to constitutional supercooling and violated *G*_*L*_/*V* criterion, catalyzing Pb‐rich cluster nucleates and growing into secondary phase. The inhomogeneous distribution of solute atoms degraded the planar interface into a rumpled or even a cellular one. The distorted interface could capture the precipitates easily, further deteriorating the surface and finally lowering the crystal quality.^[^ ^13^ ^]^

**Figure 3:**
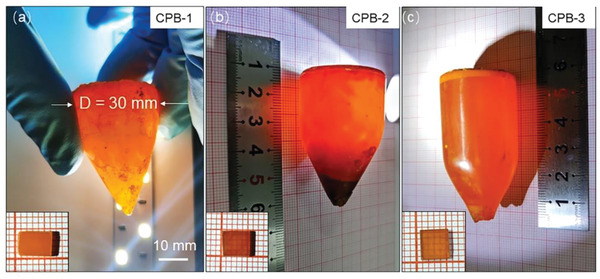
Optical images of as‐grown a) CPB‐1, b) CPB‐2, and c) CPB‐3 crystals, respectively. Insets correspond to the polished single crystal wafers cut from the as‐grown crystals.

To investigate the effects of solid–liquid interface and secondary phases on crystal quality, as well as physical properties, we grew large crystals using parameters identical to the QC‐1, 2, and 3, and denoted them as CPB‐1, 2, and 3, respectively. All resultant crystals had a diameter of 30 mm and a length of 40–70 mm, as demonstrated in **Figure** **3**a,b. Although all crystals are orange in color, they exhibit discernible optical transmission ability under nature illumination. In fact, the CPB‐1 sample is nearly opaque (inset of Figure 3a,b), suggesting dense defects are present inside it, in striking contrast to the nearly transparent CPB‐3 crystal. These observations coincide with our IR transmittance measurements in the range of 800–4000 cm^−1^, which determine transmittance of CPB‐1, ‐2, and ‐3 to be 40%, 60%, and 80% (Figure S5, Supporting Information).

**Figure 4:**
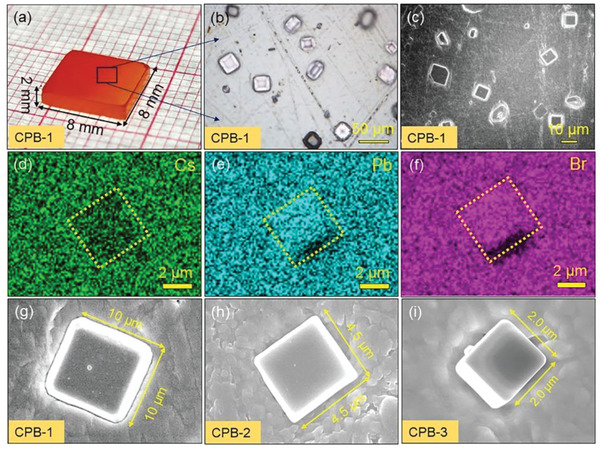
a) Polished CPB‐1 single crystal with a size of roughly 8 × 8 × 2 mm^3^. b) The representative high‐magnification optical microscope image taken on the surface. c) SEM image taken on the surface of CPB‐1. d–f) Elemental map of a precipitate in the CPB‐1 crystal by SEM‐EDS, showing the element distribution of Cs (green), Pb (blue), and Br (violet). g–i) Display of the high‐magnification SEM images taken on an isolated secondary phase at the surface of well‐polished CPB‐1, CPB‐2, and CPB‐3 crystals, respectively.

To accurately probe the possible defects in crystals, the damage layer and chemical residues on the surface of CPB were cleaned off until the surface of thin CPB wafer was highly reflective (**Figure** **4**a). To clearly visualize the distribution of secondary phase, the interfaces between the secondary phase and the matrix were exposed by chemical etching. Figure 4b,c presents the representative high‐magnification optical microscope and SEM image taken on the surface of CPB‐1, all of which show polyhedral precipitates are evenly distributed over the surrounding CsPbBr_3_ matrix. The elemental mapping on an individual polyhedral precipitate reveals it is Br rich (Figure 4d,f) and quantitative analysis indicates it has an element ratio of Cs: Pb: Br = 1: 2: 5 (Figure S6 and Table S3, Supporting Information). Figure 4g,i presents high‐magnification images of CsPb_2_Br_5_ precipitate in each crystal, revealing their lengths are 10, 4.5, and 2.0 µm. This observation is highly consistent with our previous discussion, and thereby, these CsPb_2_Br_5_ secondary phases were generated by the unstable solid–liquid interface.

It should be noted that the secondary phases were observed even before chemical corrosion, as shown in Figure S6, Supporting Information. The regular inclusions were found on the crystal surface after mechanical polishing as outlined by the red box in Figure S7a, Supporting Information. We analyzed the inclusion phase using SEM‐EDS, showing Cs: Pb: Br = 1.32: 2: 4.45 (Figure S7b, Supporting Information). This stoichiometric ratio significantly deviated from the matrix, and closes to CsPb_2_Br_5_ phase. This also proves that CsPb_2_Br_5_ secondary phase has innate defects in the material, rather than being introduced by the corrosion process. It should be noted that due to the presence of a matrix covering layer in the observed secondary phase particle area, the composition characterization of the secondary phase may deviate from the actual value.

**Figure 5:**
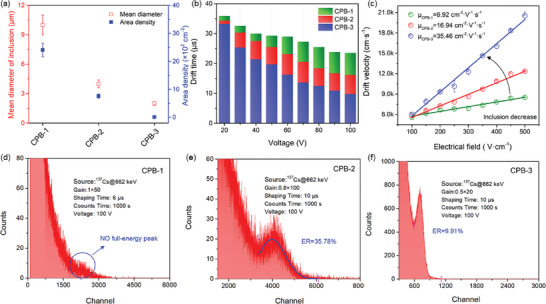
a) The average diameter and area density of secondary phase in each crystal. b) The drift time measured under different bias voltage for each crystal. c) Drift velocity as a function of electrical field for each crystal. Linear fits of the scatter point determine the electron mobility of CPB‐1, ‐2, and ‐3 crystals to be 6.92, 16.94, and 35.46 cm^2^ V^−1^ s^−1^, respectively. d–f) *γ*‐ray spectroscopy response of ^137^Cs@662 keV for CPB‐1, CPB‐2, and CPB‐3, respectively. Red circle in (d) denotes the absence of the characteristic peak of ^137^Cs@662 keV.

Given that solid–liquid interface engineering contributes significantly to the removal of unexpected secondary phases in crystal, it strongly benefits the gamma‐ray (*γ*‐ray) detection efficiency. To construct the relationship between synthesis parameters and *γ*‐ray detection efficiency, we first accurately determined the average diameter and area density for all crystals by repeating SEM measurement and extracting the length of all precipitates on the sample (Figure S8, Supporting Information). The data were plotted as a scatterplot in **Figure** **5**a, revealing that the average length and area of secondary phase in CPB‐1, 2 and 3, gradually decrease with optimized synthesis parameters. Decreasing the growth rate or increasing the temperature gradient near the growth interface can significantly reduce the size of secondary phases inclusions. The formation of secondary phase was mainly attributed to the fluctuation of S–L interface morphology. Violating *G*_*L*_/*V* criterion in the region for stable growth can lead to constitutional supercooling. It is valid to achieve a flat interface during the growth by slowing down the growth rate and/or increasing the temperature gradient. Therefore, with the optimization of the growth interface, the counts and density of secondary phases are effectively reduced.

Afterward, we assembled Sn/CsPbBr_3_/Au planar detectors and measured *α* particles response using ^241^Am @5.48 MeV radiation source. The Sn metal was selected as electrode because it is stable and has a work function (4.2 eV) lower than CsPbBr_3_. Figure S9, Supporting Information, encloses the transient pulses for all detectors under electric field, which reflects the trajectory of photo‐generated carriers. This allows us to extract the carrier drift time (*t*_r_) by truncating the preamplifier signals as shown in Figure 5b. It should be noted that *t*_r_ of each detector is in line with the density of secondary phase. For example, CPB‐3 based detector shows the shortest *t*_r_ at every single point of electric field among all detectors. In fact, CPB‐3 based detector exhibits a *t*_r_ of only ≈10 µs at 100 V voltage, nearly three times lower than that of ≈30 µs for the detector based on CPB‐1. This can be attributed to defects inducing carrier‐phonon scattering so that *t*_r_ increases with increasing secondary phases. Based on *t*_r_, the carrier mobility *µ* can be calculated by invoking the Equation (2)^[^ ^14^ ^]^:

(2) $$\mu =\frac{v_{\mathrm{dr}}}{E}=\frac{d^{2}}{V\times t_{r}}$$

where *d* denotes the detector thickness and *V* represents the bias voltage applied on the detectors. Figure 5c shows drift velocity as a function of applied electrical field. Linear fits of the scatter point determine that the electron mobilities of CPB‐1, ‐2, and ‐3 crystals are 6.92, 16.94, and 35.46 cm^2^ V^−1^ s^−1^, respectively. This finding is again consistent with our SEM observations and carrier drift time, verifying that engineering solid–liquid interface suppresses the generation of secondary phase, leading to excellent quality crystals.

The increased mobilities suggest engineering solid–liquid interface improves gamma ray detection efficiency. Figure 5d–f demonstrates the room temperature pulse height spectra of Sn/CsPbBr_3_/Au quasi‐hemispherical detectors irradiated by ^137^Cs@662 keV gamma ray at a negative bias of 100 V. Note that, the spectra under higher bias voltage were not recorded in this work because our primary experiments indicate voltage larger than 100 V negligibly improves the resolution (Figure S10, Supporting Information). It is important to note that the full energy peak of ^137^Cs@662 keV gamma ray is absent in the CPB‐1 sample, as indicated by the red circle in Figure 5d, contradictory to previous theoretical calculation. However, the characteristic peak is clearly observed by employing CPB‐2 and ‐3 crystals. Indeed, CPB‐3 detector recognizes the peak with an energy resolution of 9.91%. It is the highest value reported for CsPbBr_3_‐based detector without depth correction.

Calculations have predicted that the CsPbBr_3_ crystal can detect *γ*‐ray at room temperature with a spectral resolution surpassing those of all‐inorganic perovskite and organic–inorganic hybrid perovskite materials. However, many works of research failed to detect *γ*‐ray using CsPbBr_3_ crystal — an issue highly associated with crystal quality. We engineered and flattened the solid–liquid interface during crystal growth, markedly removing the unexpected secondary phase of CsPb_2_Br_5_ that is commonly found in the crystal. The crystal growth at small growth rate 0.5 mm h^−1^ and large temperature gradient 25.3 K cm^−1^ are optically transparent and show an exceptionally high carrier mobility of 35.46 cm^2^ V^−1^ s^−1^ at room temperature. Remarkably, it resolves the peak of ^137^Cs@662 keV *γ*‐ray at an energy resolution of 9.91%. Our results would be a major development not only because of achieving high energy resolution of *γ*‐ray using CsPbBr_3_ crystal but also because engineering solid–liquid could be applied to other materials to reach predicted properties.

## Conflict of Interest

The authors declare no conflict of interest.

## Supporting information

Supporting Information

## Data Availability

Research data are not shared.
