# Supplementary material for: High‐Performance Industrial‐Grade CsPbBr3 Single Crystal by Solid–Liquid Interface Engineering
Source: Adv Sci (Weinh). 2023 Jun 6;10(23):2302236. doi: 10.1002/advs.202302236 (PMC10427374; doi:10.1002/advs.202302236)
Supplement: Supplementary file 1 — Supporting Information [file ADVS-10-2302236-s001.pdf]

ADVANCED SCIENCE
Open Access

Supporting Information

for *Adv. Sci.*, DOI 10.1002/advs.202302236

High-Performance Industrial-Grade $CsPbBr_3$ Single Crystal by Solid–Liquid Interface Engineering

Qihao Sun, Bangzhi Ge, Bao Xiao, Fangpei Li, Leilei Ji, Ziang Yin, Jun Guo, Jia Tang, Chongjian Zhou, Wanqi Jie, Menghua Zhu and Yadong Xu**

Supplementary Material

High-Performance Industrial-Grade $CsPbBr_3$ Single Crystal by Solid-liquid Interface Engineering

Qihao Sun, Bangzhi Ge, Bao Xiao, Fangpei Li, Leilei Ji, Ziang Yin, Jun Guo, Jia Tang, Chongjian Zhou, Wanqi Jie, Menghua Zhu, and Yadong Xu

Dr. Q. Sun, Dr. B. Ge, Dr. B. Xiao, Dr. F. Li, Mr. L. Ji, Mr. Z. Yin, Dr. J. Guo, Mr. J. Tang, Prof. C. Zhou, Prof. W. Jie, Prof. M. Zhu, and Prof. Y. Xu
State Key Laboratory of Solidification Processing & Key Laboratory of Radiation Detection Materials and Devices, MIIT, School of Materials Science and Engineering, Northwestern Polytechnical University, Xi'an Shaanxi 710072, China
Email: mhzhu@nwpu.edu.cn, xyd220@nwpu.edu.cn

Q. S. and B. G. contributed equally to this work.

*Corresponding authors.

Email: xyd220@nwpu.edu.cn

Email: mhzhu@nwpu.edu.cn

Synthesis of Polycrystalline $CsPbBr_3$

High purity $PbBr_2$ (99.999%) and CsBr (99.999%) purchased from Aladdin Chemistry Co. Ltd were used as the starting materials without further purification. Stoichiometric $PbBr_2$ and CsBr with the total weight of 50–150 g was charged into a silica ampoule with a diameter of 15 mm. The silica ampoule was flame sealed under a dynamic vacuum of 5×10^{-5} Pa. The sealed ampoule was subsequently placed in a rocking furnace equipped with temperature and motor controllers. The tubes were first heated to 853 K at a rate of 50 $K\cdot h^{-1}$ and shook up for 24 hours, then slowly cooled down to 323 K at a rate of 25 $K\cdot h^{-1}$. Afterwards, orange color $CsPbBr_3$ polycrystals were obtained.

Solid-liquid interface Quenching experiments

Three CPB ingots with diameter of 15 mm were grown under different temperatures gradient and pulling rate to observe the morphology variation of the solid-liquid interface at microscale. The growth parameters for quenched CPB crystal (QC-1) were G_L = 12.1 $K\cdot cm^{-1}$ and V = 1.0 $mm\cdot h^{-1}$, comparing with QC-2 (G_L = 12.5 $K\cdot cm^{-1}$, V = 0.5 $mm\cdot h^{-1}$) and QC-3 (G_L = 20.3 $K\cdot cm^{-1}$, V = 0.5 $mm\cdot h^{-1}$). For all the three ingots, the growth was interrupted before the end by quickly removing ampoules out of furnace and quenching in the air. After cooling to room temperature, the crystals were cut longitudinally along the crystal growth direction to show the solid-liquid interface.

$CsPbBr_3$ Single Crystal Growth

CsPbBr3 single crystals were grown by a homemade dual-zone high-temperature vertical Bridgman furnace. The hot zone of the furnace was set to 873–1023 K, and the cold zone to 473 – 673 K, respectively. The temperature gradient was adjusted in the range of 10 to 30 $K\cdot cm^{-1}$ near the melting point. $CsPbBr_3$ polycrystals were transferred into a silica ampoule with carbon coating, which was sealed under a vacuum of 5×10^{-5} Pa. Then the ampoule was placed into Bridgman furnace and heated up to 853 – 873 K in 12 h. Subsequently, the ampoule was slowly moved downwards to the cold zone with a rate of 0.5–1.0 $mm\cdot h^{-1}$. When the crystallization process was finished, the furnace was cooled down to room temperature with a rate of 4 – 10 $K\cdot h^{-1}$.

Defect Visualization

$CsPbBr_3$ crystal were cut from the as-grown ingot by a single wire saw. The defect visualization processing involves two procedures, polishing and etching, respectively. CPB wafers were roughly polished by 3000#–5000# abrasive paper and velvet

polishing pad, and then finely polished with micron magnesium oxide particles (50 nm) EtOH solution. Subsequently, the wafer was chemically etched using HBr and EtOH mixed solution with a volume fraction of 10–15% for 5–30 mins at room temperature. Finally, the etching solution on the wafer was removed by toluene, prior to surface observation.

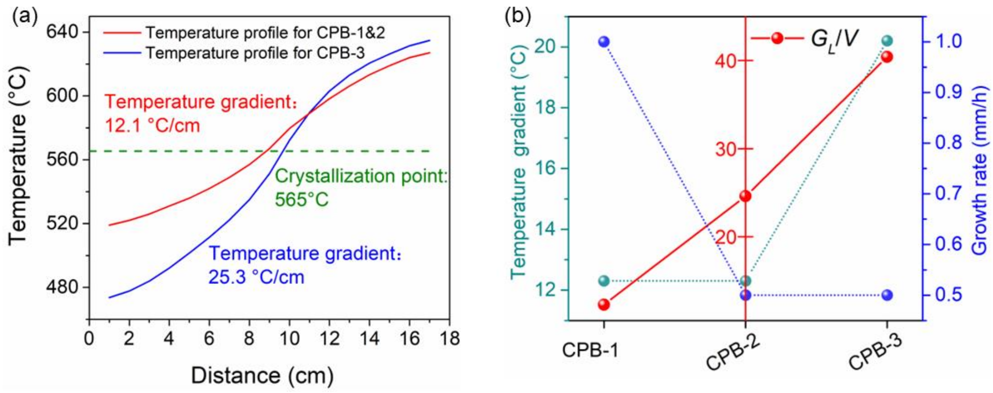

Figure S1. (a) The temperature profiles for $CsPbBr_3$ crystal along axis of furnace. (b) The ratio of G_L/V for CPB-1, CPB-2 and CPB-3.

Table S1. the parameters for growing $CsPbBr_3$ crystal

Crystal	Growth rate (V)	Gemperature gradient (G_L)	G_L/V
CPB-1	1.0 mm·h^{-1}	12.1 K·cm^{-1}	12.1
CPB-2	0.5 mm·h^{-1}	12.1 K·cm^{-1}	24.2
CPB-3	0.5 mm·h^{-1}	25.3 K·cm^{-1}	50.6

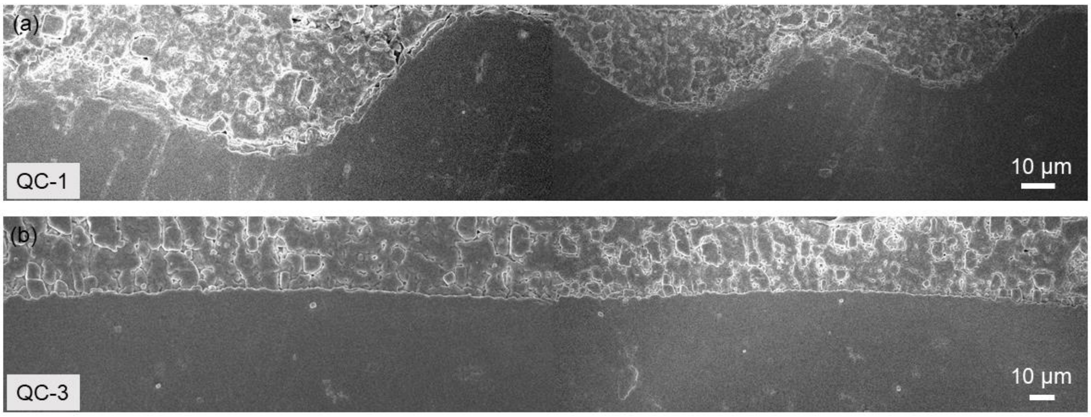

Figure S2. SEM images taken on the solid-liquid interface of (a) QC-1 and (b) QC-3.

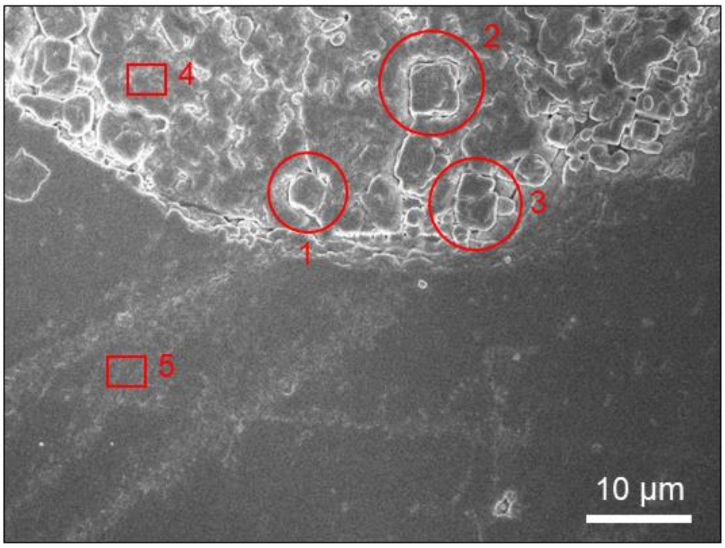

Figure S3. The typical SEM morphology of QC-1 solid-liquid growth interface

Table S1. Concentration at different sites near quenched solid-liquid interface

Elements	Position 1 (at%)	Position 2 (at%)	Position 3 (at%)	Position 4 (at%)	Position 5 (at%)
Cs	13.03	13.83	13.99	20.12	20.11
Pb	24.94	24.65	23.54	20.19	20.16
Br	62.03	61.52	62.47	59.69	59.73
Total	100	100	100	100	100

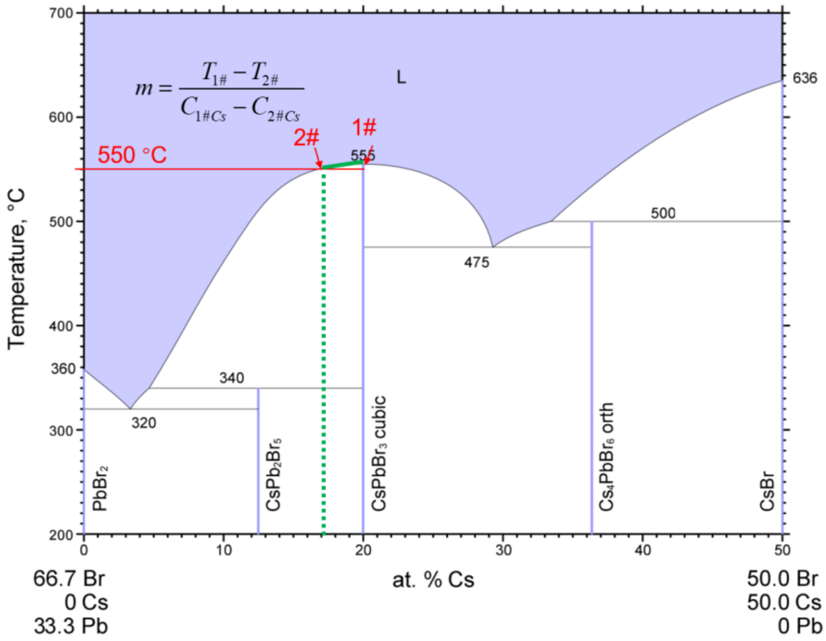

Figure S4. Pseudo binary phase diagram of $PbBr_2$-CsBr

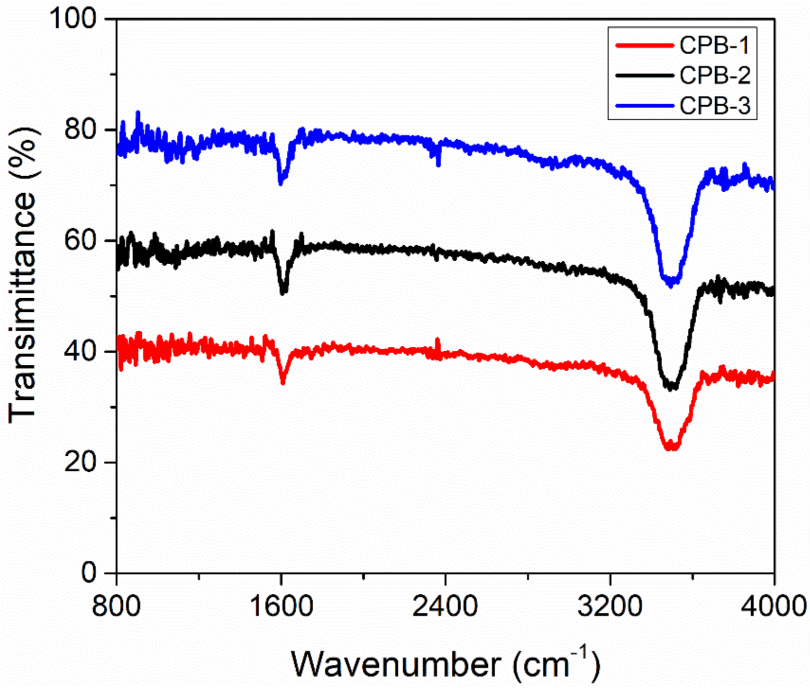

Figure S5. Infrared transmission spectrum of $CsPbBr_3$ wafers at room temperature.

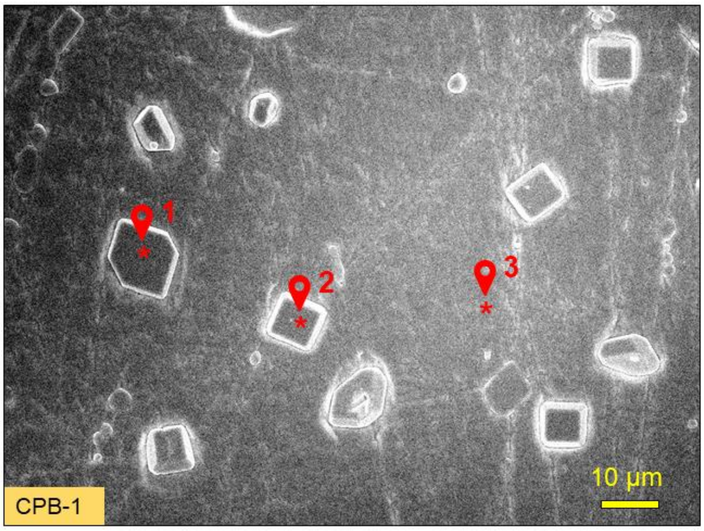

Figure S6. The typical CPB wafer surface after selective etching and EDS point selection.

Table S3. The atomic percentage at different sites by EDS micro-area test

Elements	Position 1	Position 2	Position 3
Cs	13.03	13.83	20.11
Pb	24.94	24.65	20.16
Br	62.03	61.52	59.73
Total	100	100	100

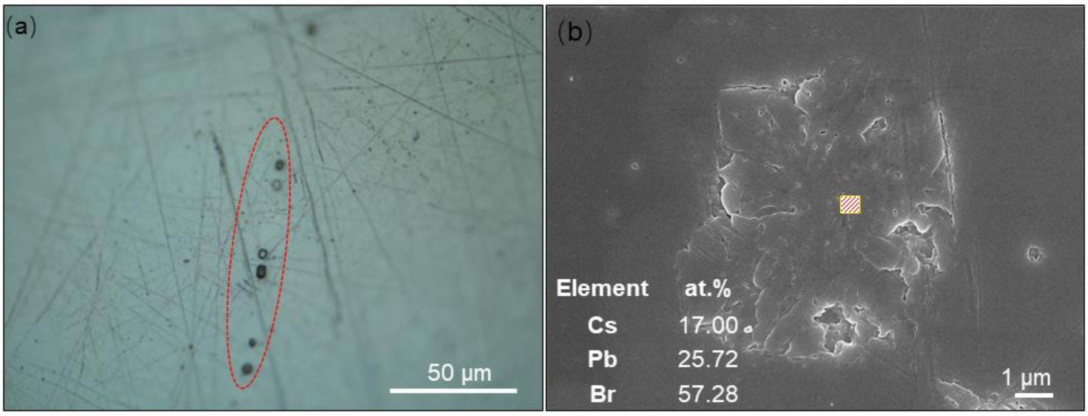

Figure S7. (a) The optical microscope image of $CsPbBr_3$, the red wire frame marks the SP particles. (b) The SEM image and EDS component characterization results of SP particles.

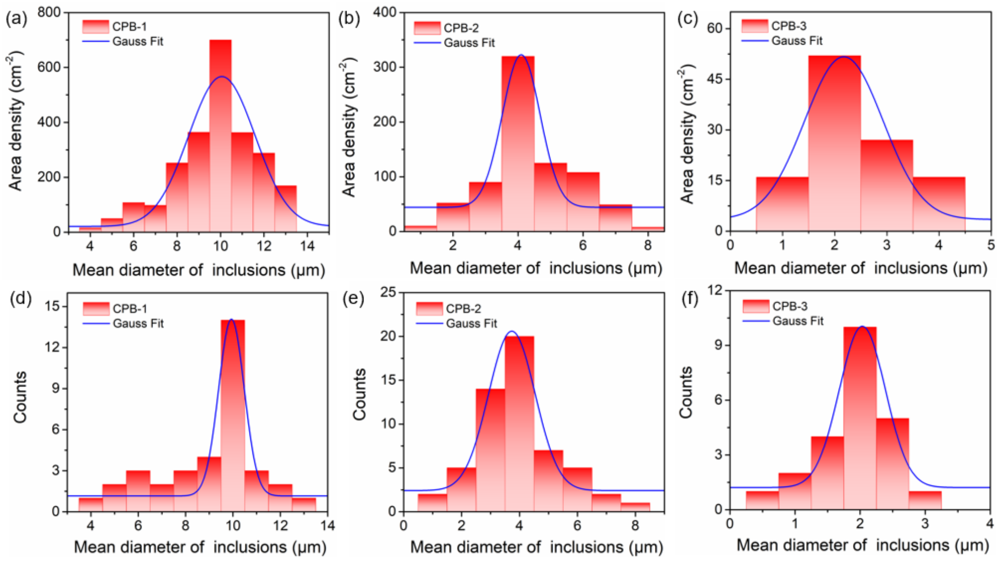

Figure S8. The histogram of area density of SP inclusion with Gaussian distribution (a) CPB-1, (b) CPB-2, and (c) CPB-3. The histogram of size of the inclusion with Gaussian distribution (d) CPB-1, (e) CPB-2, and (f) CPB-3.

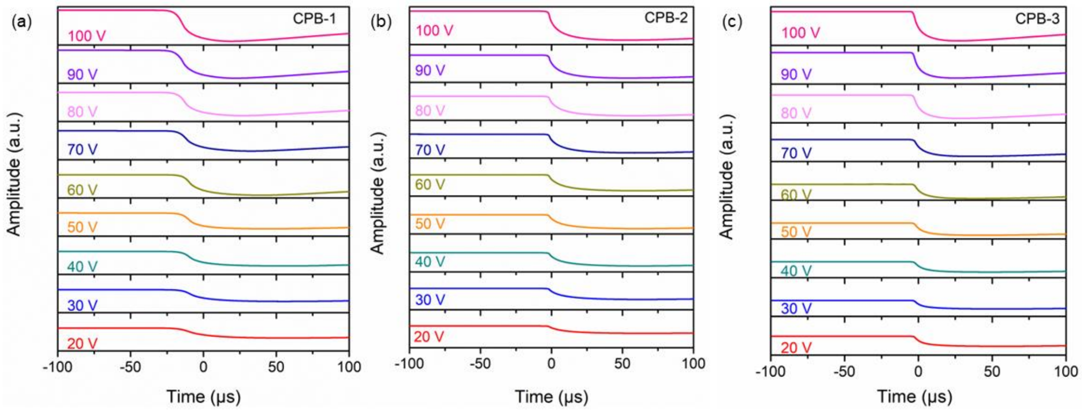

Figure S9. α particles induced pulse shapes under the same electric field strength: (a) CPB-1, (b)CPB-2, (c) CPB-3.

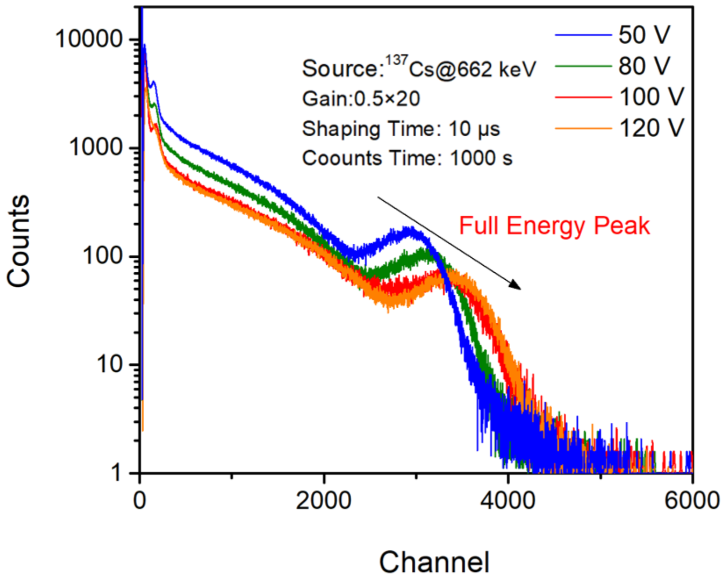

Figure S10. Response energy spectrum of 662 keV@^{137}Cs γ ray under different applied voltages of CPB-3
